# The FoxO3 gene and cause‐specific mortality

**DOI:** 10.1111/acel.12452

**Published:** 2016-04-13

**Authors:** Bradley J. Willcox, Gregory J. Tranah, Randi Chen, Brian J. Morris, Kamal H. Masaki, Qimei He, D. Craig Willcox, Richard C. Allsopp, Stefan Moisyadi, Leonard W. Poon, Beatriz Rodriguez, Anne B. Newman, Tamara B. Harris, Steven R. Cummings, Yongmei Liu, Neeta Parimi, Daniel S. Evans, Phil Davy, Mariana Gerschenson, Timothy A. Donlon

**Affiliations:** ^1^ Department of ResearchKuakini Medical CenterHonoluluHI96817USA; ^2^ Department of Geriatric MedicineJohn A. Burns School of MedicineUniversity of HawaiiHonoluluHI96817USA; ^3^ California Pacific Medical Center Research InstituteSan FranciscoCA94107USA; ^4^ School of Medical Sciences and Bosch InstituteUniversity of SydneySydneyNSW2006Australia; ^5^ Department of Human WelfareOkinawa International UniversityGinowanOkinawa901‐2701Japan; ^6^ Institute for Biogenesis ResearchUniversity of HawaiiHonoluluHI96813USA; ^7^ Institute of GerontologyUniversity of GeorgiaAthensGA30602USA; ^8^ Department of EpidemiologyUniversity of PittsburghPittsburghPA15261USA; ^9^ Laboratory of NeurogeneticsIntramural Research ProgramNational Institute on AgingBethesdaMD20892USA; ^10^ Division of Public Health SciencesWake Forest School of MedicineWinston‐SalemNC27157USA; ^11^ Department of Cell & Molecular BiologyUniversity of HawaiiHonoluluHI96813USA

**Keywords:** *FOXO*3, heart disease, longevity, mortality

## Abstract

The *G* allele of the *FOXO3* single nucleotide polymorphism (SNP) *rs2802292* exhibits a consistently replicated genetic association with longevity in multiple populations worldwide. The aims of this study were to quantify the mortality risk for the longevity‐associated genotype and to discover the particular cause(s) of death associated with this allele in older Americans of diverse ancestry. It involved a 17‐year prospective cohort study of 3584 older American men of Japanese ancestry from the Honolulu Heart Program cohort, followed by a 17‐year prospective replication study of 1595 white and 1056 black elderly individuals from the Health Aging and Body Composition cohort. The relation between *FOXO3* genotype and cause‐specific mortality was ascertained for major causes of death including coronary heart disease (CHD), cancer, and stroke. Age‐adjusted and multivariable Cox proportional hazards models were used to compute hazard ratios (HRs) for all‐cause and cause‐specific mortality. We found *G* allele carriers had a combined (Japanese, white, and black populations) risk reduction of 10% for total (all‐cause) mortality (HR = 0.90; 95% CI, 0.84–0.95; *P *=* *0.001). This effect size was consistent across populations and mostly contributed by 26% lower risk for CHD death (HR = 0.74; 95% CI, 0.64–0.86; *P* = 0.00004). No other causes of death made a significant contribution to the survival advantage for *G* allele carriers. In conclusion, at older age, there is a large risk reduction in mortality for *G* allele carriers, mostly due to lower CHD mortality. The findings support further research on *FOXO3* and FoxO3 protein as potential targets for therapeutic intervention in aging‐related diseases, particularly cardiovascular disease.

## Introduction

The aging of the world's population has enormous implications for society, social insurance programs, and health care (National Institute on Aging, 2007; US Department of Health and Human Services, 2012). Thus, understanding how humans can age more healthfully is of vital importance. Genetic factors that affect the rate of aging and/or risk for age‐related diseases are important, as they account for approximately one‐third of the variability in human lifespan (Brooks‐Wilson, 2013; Shadyab & LaCroix, 2015). However, despite almost three decades since the first finding of longevity‐associated genotypes in long‐lived persons (Takata *et al*., 1987), the effects on longevity of only the apolipoprotein E gene (*APOE*) and the forkhead box O‐3 (FoxO3) gene (*FOXO3*) have been consistently replicated across multiple, diverse human populations (Flachsbart *et al*., 2009; Pawlikowska *et al*., 2009; Murabito *et al*., 2012; Brooks‐Wilson, 2013; Morris *et al*., 2015; Shadyab & LaCroix, 2015).

FoxO3 is an evolutionarily conserved transcription factor in the insulin signaling pathway (Kenyon, 2010). It regulates expression of genes controlling a multitude of processes that could enhance health and lifespan (Willcox *et al*., 2008; Kenyon, 2010; Eijkelenboom & Burgering, 2013). A previous study of American men of Japanese ancestry was the first to find an association of three single nucleotide polymorphisms (SNPs) of *FOXO3* with human longevity, the strongest SNP being *rs2802292* (Willcox *et al*., 2008). The association was replicated in 11 other independent studies of populations of diverse ancestry (Bao *et al*., 2014). A meta‐analysis found these and two other *FOXO3* SNPs retained statistical significance, the strongest association with longevity involving the original *G* allele of *rs2802292* in men (odds ratio (OR), 1.54; 95% confidence intervals (CIs), 1.33–1.67) (Bao *et al*., 2014). A meta‐analysis of genomewide association studies of longevity yielded an OR of 1.17 (*P *=* *1.9 × 10^−10^) for this allele (Broer *et al*., 2015). The effect size has not been well quantified in terms of mortality risk over time. The mechanisms by which the protective allele(s) reduce mortality to promote human longevity are also not known. Identifying the cause of death in longevity‐allele carriers vs. noncarriers may provide clues as to why *FOXO3* SNPs strongly protect against mortality.

We hypothesized that the longevity‐associated *FOXO3* genotype would be associated with a sizable risk reduction for mortality and with one or more major age‐associated clinical causes of death, such as coronary heart disease (CHD), cancer, and stroke. To test this hypothesis we utilized an extensive, prospectively collected dataset from our long‐lived cohort of American men of Japanese ancestry, well characterized for aging phenotypes, drawn from the Honolulu Heart Program (HHP) prospective cohort study (Kagan, 1996). We genotyped this study population to prospectively assess the following: (i) the effect size of the protective (longevity‐associated) *FOXO3* genotype on total (all‐cause) mortality in 17 years of follow‐up; and (ii) the effect of the protective *FOXO3* genotype on cause‐specific mortality. We then attempted a replication of major findings in a suitable cohort of elderly white and black Americans of both sexes in the Health Aging and Body Composition (ABC) cohort study, which had 17 years of follow‐up.

## Results

### Study participants

The baseline characteristics of each cohort of subjects (Japanese, white, and black) are shown in Table 1. HHP study participants tended to be older and had higher prevalence of hypertension, diabetes, and CHD than Health ABC participants. There was a higher percentage of *G* allele carriers in the Health ABC population, particularly in blacks, in whom the *G* allele was the common allele.

**Table 1:** Characteristics of the three study cohorts of older Americans

Parameter	Japanese (*n* = 3584)	Whites (*n* = 1794)	Blacks (*n* = 1281)
Age (years)	77.7 ± 4.6	73.8 ± 2.9	73.4 ± 2.9
Body mass index (kg m^−2^)	23.5 ± 3.2	26.5 ± 4.1	28.6 ± 5.4
History of ever smoking (%)	62.4	56.8	55.2
Alcohol (3+ drinks per week) (%)	40.3	23.3	9.6
Exercise regularly (%)	68.3	49.7	31.2
Glucose (g dL^−1^)	113 ± 29.5	100.8 ± 27.3	109.3 ± 41.8
Total cholesterol (mg dL^−1^)	189.8 ± 33.1	201.3 ± 37.4	204.9 ± 40.1
CHD (%)^a^	20.6	13.1	13.7
Stroke (%)^a^	4.5	1.4	3.7
Cancer (%)^a^	15.6	24.5	11.0
Diabetes (%)^a^	28.6	10.7	21.0
Hypertension (%)^a^,^b^	73.6	48.7	62.9
*FOXO3 G* allele frequency (%)	27.7	36.5	73.2
*FOXO3 G* allele carrier (%)^c^	47.0	58.4	92.7

Americans of Japanese ancestry tended to be older, had more prevalent CHD and diabetes, and lower frequency of the longevity‐associated *FOXO3* G allele at the baseline examination.

a Prevalent disease (not age‐adjusted) was defined and detected based on respective study surveillance programs: CHD: any myocardial infarction, bypass surgery, angioplasty, other heart surgery, or ECG evidence; Diabetes: fasting glucose ≥ 126 mg mL^−1^ or 2‐h glucose ≥ 200 mg mL^−1^, or on diabetes medication.

b Participants were considered to have hypertension if they had a systolic blood pressure of ≥ 140 mmHg and/or diastolic blood pressure of ≥ 90 mmHg or were on antihypertensive medication.

c Carriers were persons who had at least one *G* allele of *FOXO3* SNP *rs2802292*. Shown are homozygotes and heterozygotes combined.

### Meta‐analysis of mortality (all‐cause)

**Figure 1:**
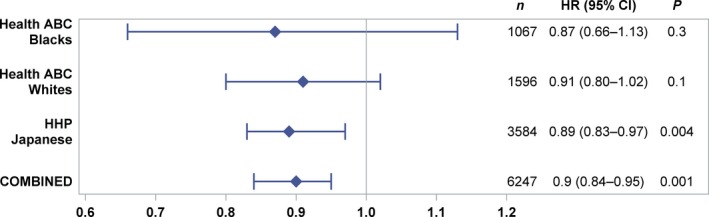
Meta‐analysis of all‐cause mortality for *G* allele carriers. Hazard ratios (HRs) for mortality over prospective follow‐up periods of 17 years for the cohorts of Americans of black (Health ABC) and white (Health ABC) individuals and 17 years for Americans of Japanese (Honolulu Heart Program (HHP)) ancestry, utilizing a dominant model. Tests for heterogeneity for all populations combined were *P *=* *0.96, demonstrating that the populations are appropriate for a combined meta‐analysis. During the 17‐year prospective follow‐up, *G* allele carriers (*FOXO3* SNP *rs2802292*) had HR that ranged from 0.87 (blacks) to 0.91 (whites) and a combined risk reduction of 10% for total mortality (HR = 0.90; 95% CI, 0.84–0.95; *P *=* *0.001).

Cox proportional hazards models were used to generate hazard ratios (HRs) over a 17‐year prospective follow‐up for black (Health ABC), white (Health ABC), and Japanese (HHP) ancestry. Tests for heterogeneity for all populations combined yielded *P *=* *0.96, demonstrating that the populations could be used for a combined analysis, utilizing a dominant model. During follow‐up, mortality risk for *G* allele carriers ranged from 0.87 (blacks) to 0.91 (whites) with a combined (Japanese, white, and black populations) risk reduction of 10% for total mortality (HR = 0.90; 95% CI, 0.84–0.95; *P *=* *0.001) (Fig. 1). Tests of model proportionality were met.

### Total (all‐cause) mortality risk for carriers of the *FOXO3 G* allele

In the HHP cohort, risk of death over a 17‐year prospective follow‐up period for carriers (vs. noncarriers as baseline risk group) of the longevity‐associated *G* allele of *FOXO3* (*rs2802292*) in Japanese American men, utilizing a multivariable Cox regression model, demonstrated an 11% risk reduction (HR 0.89; 95% CI: 0.83–0.97; *P *=* *0.004) for all‐cause mortality (Table 2A). Five important risk factors for mortality in older persons did not substantially alter mortality risk, suggesting that they are not in the causative pathway between *FOXO3* variation and mortality in older Japanese males.

**Table 2:** All‐cause mortality risk for carriers of the *FOXO3* *G* allele in multivariable risk factor model. (A) Honolulu Heart Program cohort. (B) Health ABC cohort

(A)		
Risk factor	HR (95% CI)	*P*
Age (years)	1.11 (1.10–1.12)	< 0.001
Fasting glucose (per 10 mg dL^−1^)	1.06 (1.05–1.08)	< 0.001
Smoking history^a^ (per 10 pack‐years)	1.04 (1.03–1.05)	< 0.001
Systolic blood pressure (per 10 mm Hg)	1.00 (0.98–1.02)	0.84
Fasting total cholesterol (per 10 mg dL^−1^)	0.98 (0.97–0.99)	0.007
Body mass index (per kg m^−2^)	0.95 (0.94–0.97)	< 0.001
*FOXO3* *G* allele carrier (age‐adjusted)	0.89 (0.83–0.97)	0.004
*FOXO3* *G* allele carrier^b^ (age‐ and risk factor‐adjusted)	0.86 (0.79–0.93)	< 0.001

(B)				
	Blacks		Whites	
Risk factor	HR (95% CI)	*P*	HR (95% CI)	*P*
Age (years)	1.06 (1.03–1.09)	< 0.0001	1.12 (1.09–1.14)	< 0.0001
Fasting glucose (per 10 mg dL^−1^)	1.03 (1.02–1.05)	< 0.0001	1.06 (1.04–1.08)	< 0.0001
Smoking history (ever smoking)	1.26 (1.08–1.46)	0.003	1.37 (1.2–1.55)	< 0.0001
Systolic blood pressure (per 10 mmHg)	1.06 (1.02–1.1)	0.002	1.01 (0.98–1.05)	0.39
Fasting total cholesterol (per 10 mg dL^−1^)	0.97 (0.95–0.98)	0.0002	0.97 (0.96–0.99)	0.001
Body mass index (per kg m^−2^)	0.97 (0.96–0.98)	< 0.0001	0.98 (0.97–1.00)	0.047
FOXO3 *G* allele carrier (age‐adjusted)	0.87 (0.66–1.13)	0.29	0.91 (0.91–1.02)	0.063
FOXO3 *G* allele carrier^c^ (age‐ and risk factor‐adjusted)	0.91 (0.69–1.2)	0.51	0.91 (0.81–1.03)	0.15

(A) Risk of death over a 17‐year follow‐up period for carriers vs. noncarriers of longevity‐associated *G* allele of *FOXO3* (*rs2802292*) in HHP population of Japanese Americans. (B) Risk of death over a 17‐year follow‐up period for carriers vs. noncarriers of longevity‐associated *G* allele of *FOXO3* (*rs2802292*) in Health ABC cohorts.

a Number of packs smoked per day × number of years smoked (pack‐years).

b Multivariable Cox regression model for subjects with complete risk factor data (*n* = 3200; total deaths = 2345). HR = hazard ratio for all‐cause mortality.

c Multivariable Cox regression model for subjects with complete risk factor data. HR = hazard ratio for all‐cause mortality.

In the Health ABC cohort, a similar analysis of risk of death over 17 years of follow‐up demonstrated a similarly large (9%) risk reduction for all‐cause mortality in whites (HR 0.91; 95% CI: 0.80–1.02; *P *=* *0.06) and 13% in blacks (HR 0.87; 95% CI: 0.66–1.13; *P *=* *0.29), respectively(Table 2B). When entering five important mortality risk factors for mortality at older ages into the model, there was a reduction in statistical significance and effect size (HR) in whites and blacks, suggesting that one or more risk factor variables are in the causative pathway between *FOXO3* variation and mortality in these populations.

### Specific causes of death and HR for mortality (age‐adjusted)

In the HHP cohort, the *G* allele conferred a highly statistically significant protective effect against CHD mortality in Japanese American men (Table 3A). Hazard ratio in *G* (longevity allele) carriers (HR: 0.75; 95% CI 0.63–0.90; *P *=* *0.001) was 25% lower than noncarriers.

**Table 3:** Specific causes of death and hazard ratio, age‐adjusted, for carriers of the *FOXO3 G* allele. (A) Honolulu Heart Program (HHP) cohort. (B) Health ABC cohort

(A)			
Cause of death	No. of deaths	HR^a^ (95% CI)	*P*‐value
Cancer	546	1.01 (0.85–1.19)	0.93
CHD (coronary heart disease)	524	0.75 (0.63–0.90)	0.001
Stroke	315	0.97 (0.77–1.21)	0.76
Dementia	221	1.01 (0.78–1.32)	0.93
Other cardiovascular disease (CVD)	213	0.85 (0.65–1.11)	0.23
Infectious disease	188	0.91 (0.68–1.21)	0.52
Chronic obstructive pulmonary disease (COPD)	117	0.83 (0.57–1.19)	0.31
Renal failure	45	0.86 (0.48–1.55)	0.61
GI (gastrointestinal disease)	39	1.22 (0.65–2.30)	0.54
Other deaths	480	0.86 (0.72–1.03)	0.09

(B)									
	Whites			Blacks			Combined		
Cause of death^a^	*n*	HR^a^ (95% CI)	*P*	*n*	HR^a^ (95% CI)	*P*	*n*	HR (95% CI)	*P*
	927			687			1614		
Cancer	220	0.87 (0.67–1.14)	0.31	179	1.67 (0.82–3.41)	0.16	399	0.97 (0.76–1.24)	0.81
CHD	224	0.76 (0.58–0.98)	0.04	140	0.61 (0.35–1.04)	0.07	364	0.73 (0.57–0.92)	0.0088
Stroke	80	0.87 (0.56–1.35)	0.53	67	0.55 (0.26–1.15)	0.11	147	0.78 (0.53–1.15)	0.21
Other CVD	41	0.96 (0.52–1.8)	0.91	47	1.04 (0.32–3.38)	0.95	88	0.98 (0.57–1.7)	0.96
COPD	42	0.82 (0.44–1.5)	0.51	29	2.07 (0.28–15.3)	0.47	71	0.91 (0.52–1.6)	0.75
Infection	36	1.21 (0.62–2.4)	0.58	24	0.54 (0.16–1.84)	0.33	60	1.03 (0.56–1.91)	0.92
Renal failure	17	0.61 (0.24–1.59)	0.32	25	aN/A	N/A	42	1.01 (0.45–2.26)	0.98
GI	7	0.88 (0.2–3.94)	0.87	9	0.46 (0.05–3.85)	0.47	16	0.72 (0.2–2.59)	0.61
Other	260	1.11 (0.87–1.43)	0.40	167	0.69 (0.4–1.18)	0.18	427	1.02 (0.81–1.29)	0.84

a HR shown is for carriers of the *G* allele. (A) Number of deaths are all deaths in each disease category from *n* = 2688 total deaths in HHP subjects (mean age 77.7 ± 4.6 years at baseline) followed up for 17 years. Cause of death was coded according the International Classification of Diseases (ICD) system (version 8) by an expert morbidity and mortality committee after comprehensive review of multiple data sources including death certificates, hospital discharge records and interviews with physicians, among other data. All the study participants were ascertained for vital status at the end of study; in only 17 (1.3%) deaths could a cause not be ascertained. There was a large (25%) risk reduction in CHD mortality. Bonferroni correction of CHD death yielded *P *=* *0.01. (B) Number of deaths are all deaths in each disease category from *n* = 1614 total deaths in Health ABC subjects followed up for 17 years. Surveillance for cause of death was similar to HHP methodology. There was a large (24%) risk reduction in CHD mortality for whites (HR: 0.76; 0.58–0.98; *P *=* *0.036) and a 39% risk reduction for blacks (HR: 0.61; 95% CI 0.35–1.04; *P *=* *0.068 for trend). White males and females were aged 73.8 ± 2.9 years at baseline. Black males and females were aged 73.4 ± 2.9 years at baseline. Bonferroni correction of CHD death yielded *P *=* *0.32 for blacks and *P *=* *0.08 for whites. CVD, cardiovascular disease; GI, gastrointestinal.

In the Health ABC cohort, there was a similarly large (24%) risk reduction in CHD mortality for whites (HR: 0.76; 95% CI 0.58–0.98; *P *=* *0.036) and even larger for blacks (39%; HR 0.61; 95% CI 0.35–1.04; *P *=* *0.068 for trend; Table 3B). While certain other diseases (such as cancer in whites and stroke in blacks) showed lower HRs for mortality, none reached statistical significance.

### Meta‐analysis of CHD mortality

**Figure 2:**
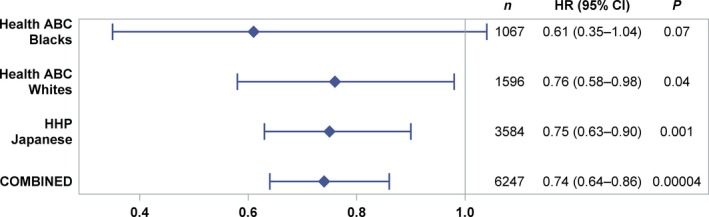
Meta‐analysis of CHD mortality. Hazard ratios (HRs) for CHD mortality over prospective follow‐up periods of 17 years for the cohorts of Americans of black (Health ABC; *n* = 140 CHD deaths), white (Health ABC; *n* = 224 CHD deaths) and 17 years for Americans of Japanese (Honolulu Heart Program (HHP; *n* = 524 CHD deaths)) ancestry, utilizing a dominant model. Tests for heterogeneity for all populations combined yielded *P *=* *0.75, demonstrating that the populations could be used for a combined analysis, utilizing a dominant model. CHD mortality risk for *G* allele carriers ranged from 0.61 (blacks) to 0.76 (whites) with a combined (Japanese, white and black populations) risk reduction of 26% for total mortality (HR = 0.74; 95% CI, 0.64–0.86; *P *=* *0.00004). Tests of model proportionality were met.

Cox proportional hazards models were used to generate hazard ratios over the 17‐year prospective follow‐up period for individuals of black (Health ABC), white (Health ABC), and Japanese (HHP) ancestry. Tests for heterogeneity for all populations combined yielded *P *=* *0.75, demonstrating that the populations could be used for a combined analysis, utilizing a dominant model. During follow‐up, CHD mortality risk for *G* allele carriers ranged from 0.61 (blacks) to 0.76 (whites) with a combined (Japanese, white, and black populations) risk reduction of 26% for total CHD mortality (HR = 0.74; 95% CI, 0.64–0.86; *P *=* *0.00004) (Fig. 2). Tests of model proportionality were met.

### *FOXO3* genotype and age at death

**Figure 3:**
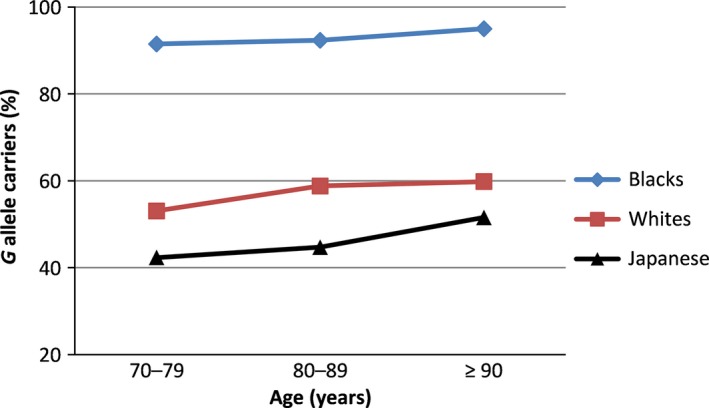
*FOXO3* *G *allele carrier status (as % of cohort) by attained age. Percentage of carriers of the longevity‐associated* FOXO3* *G* allele of SNP *rs2802292* increased with age in the pooled population (*P* < 0.0001; logistic regression; adjusted for race).

Carrier status for the protective *G* allele increased from age in the 70s to age of ≥ 90 years, consistent with protection against mortality for carriers in the three populations (Fig. 3).

### Serum TNF‐α level by *FOXO3 G* allele carrier status

In a pilot analysis of HHP Japanese men, carriers of the longevity‐associated *G* allele of *FOXO3* SNP *rs2802292* had lower blood levels of the inflammatory cytokine TNF‐α (Table S1).

## Discussion

In this, the first large, comprehensive, multiethnic prospective cohort study to quantify the risk reduction associated with the minor allele *(G)* of the *FOXO3* longevity‐associated genotype, we demonstrated a large (10%) protective effect against all‐cause mortality and 26% for CHD mortality over 17 years of follow‐up. The protective effect was, moreover, observed in three genetically different populations. Our study contrasts with the vast majority of prior investigations of *FOXO3* variants and longevity, which have been case:control studies that did not quantify risk over time, but rather simply tested for association with an outcome.

The magnitude of the impact of an *absence* of the *FOXO3* *G* allele was comparable to the increase in risk of death from smoking a pack of cigarettes a day for 25 years in Japanese men. In black males and females, it was equivalent to having a 20 mmHg higher systolic blood pressure, and in white men and women to a 20 mg dL^−1^ elevation in fasting blood glucose.

Of particular interest, in multivariable models of mortality risk factors, five of the top risk factors for mortality at older ages (i.e. smoking, hypertension, fasting glucose, low BMI, low cholesterol) had little influence on the effect on mortality of *FOXO3* genotype in Japanese men. In the Health ABC cohort of white and black men and women, there did appear to be mediator variables, in that the effect was partially attenuated when risk variables were entered into a multivariable model. The data suggest a possible mediator role for hypertension, which we found to be less prevalent in middle‐aged women carrying the *FOXO3 G* allele (Morris *et al*., 2015).

Although we found a lower risk for stroke in all three populations, this did not reach statistical significance. The only prior studies by others of *FOXO3* genotype and cardiovascular disease led to conflicting results. One, a 4‐year prospective cohort study of Dutch men and women aged ≥ 85 years found that a particular *FOXO3* haplotype conferred an increased risk for incident stroke and also an increased risk of mortality from CHD, stroke, and all‐cause (total) mortality (Kuningas *et al*., 2007). Another investigation, a case:control study of Danish men and women aged ≥ 90 years, failed to find an association of *FOXO3* genotype with CHD or stroke (Soerensen *et al*., 2015). A case:control study of Han Chinese found no relation with CHD (Zhao *et al*., 2014), while two other case:control studies found *FOXO3* longevity‐allele carriage was associated with lower CHD prevalence (Japanese American males) (Willcox *et al*., 2008) and with lower CHD and stroke mortality (white males and females) (Pawlikowska *et al*., 2009).

Although *FOXO3* is a tumor suppressor (Lam *et al*., 2013; Fruman & Rommel, 2014), we could find only one study that reported an association of *FOXO3* genotype with all‐cancer mortality (Pawlikowska *et al*., 2009) and another study in which a borderline association with cancer prevalence was found (Willcox *et al*., 2008). Both were case:control studies of longevity‐associated alleles. The current study found no detectable protection against cancer mortality, although larger studies of particular cancer subtypes may be useful.

It should, however, be noted that the prior studies were limited by small sample sizes, short follow‐up time, a case:control study design, monoethnic populations, and/or very old age of the study subjects. The current study did not suffer from these challenges. Therefore, while a contributory role for other diseases and disease processes cannot be ruled out, protection against CHD mortality seems paramount.

Based on studies of FoxO3 in model organisms we suspect immune dysregulation may be particularly important. In aging humans, adaptive immunity decreases, leading to immunosenescence, but innate immunity increases, leading to a pro‐inflammatory phenotype termed ‘inflamm‐aging’ (Franceschi *et al*., 2007; Peng, 2007; Salminen *et al*., 2008). FoxO3 inhibits the production of several inflammatory cytokines linked to human aging. These include interleukin‐2 (Oh *et al*., 2011), interleukin‐6 (Dejean *et al*., 2009), and TNF‐α (Lee *et al*., 2013), each of which has been linked to both aging and cardiovascular disease.

In support of this premise, the minor allele of *FOXO3* SNP *rs12212067* (which is in linkage disequilibrium with *rs2802292* and could act as a proxy) was associated with a milder clinical course of seemingly unrelated inflammatory conditions (Crohn's disease and rheumatoid arthritis) (Lee *et al*., 2013). Minor allele carriage limited inflammatory responses in monocytes through TGF‐β1‐induced reduction of pro‐inflammatory cytokines, including TNF‐α. It also increased production of anti‐inflammatory cytokines. Our finding of lower TNF‐α in *FOXO3* *G* allele carriers is consistent with protection against inflammation and thence CHD. More work is needed to confirm a major influence of reduced inflammation conferred by *FOXO3* genotype in protection against CHD, as an explanation for its ability to confer increased lifespan.

Interestingly, the *G* allele is the ancestral allele as it is the most prevalent allele in African black populations and it persists as the common allele in the American black population (dbSNP, 2016). A question arises as to why the nonprotective (*T*) allele has become the common allele in whites and Asians. This is likely the result of population bottle necks that have pruned the *G* allele frequencies in “out of Africa” populations. This process could have fixed the *rs2802292 G* allele in a haplotype that contains a functional variant, such as one that results in better old age health. For most of human history infectious disease, of which inflammation is a major component, was likely the major cause of death (Finch, 2010). Perhaps *TT* homozygotes may not be able to defend against a high inflammatory load, which can lead to sepsis (among other potentially fatal maladies), and thus, fewer of these survived to pass on *T* alleles. It is plausible that there might be an unknown antagonistic pleiotropic effect where the allele protects early in life and becomes a risk later in life (Blagosklonny, 2010). There are examples of antagonistic pleiotropy involving the FoxO3 gene in model organism studies (Shen & Tower, 2010). As yet, we do not know if *FOXO3* genotype protects against mortality at young or middle age, only that it protects against mortality in later life, as no studies have assessed the effects of *FOXO3* genotype over the adult life course in humans. There are also several other plausible explanations of why the *G* allele has persisted as the common allele in blacks but has decreased in frequency in white and Asian populations. FoxO is critical in maintaining stem cell populations (Miyamoto *et al*., 2007). Perhaps human populations have only recently been able to survive long enough for the beneficial effects of the *FOXO3* *G* allele on stem cell populations to be realized. Another potential explanation may be the fact that FoxO3 regulates stress resistance and protects against ultraviolet damage (Yang *et al*., 2006). Ultraviolet radiation would have been a major biological stressor in African populations. Future work may be able to answer such questions.

Our study has several strengths, including rigorous ascertainment of cause of death, large sample size that was adequate for detection of dominant genetic effects of *FOXO3* on cause‐specific mortality, and replication of the main findings across cohorts. Another major strength is the prospective, population‐based cohort design, with virtually complete mortality ascertainment in near‐extinct cohorts. This eliminated recall bias concerning prevalent diseases and risk factors and also allowed us to quantify cause‐specific mortality risk over time. The relative homogeneity of the HHP population was also an advantage, and prior work has found no evidence of a population stratification artifact in this cohort (Willcox *et al*., 2008). Population substructure was also controlled for in the Health ABC population. The replication we saw in a methodologically similar cohort of elderly white and black Americans demonstrates that the findings are robust and generalizable to other genetic populations.

## Summary and conclusions

The present study is the first to quantify the protection against mortality in a large, well‐powered, prospective cohort study of populations of contrasting ethnicities, and to demonstrate that CHD is the main clinical cause of death driving the strong survival advantage seen in carriers of the major longevity‐associated *FOXO3* allele. As CHD is the primary cause of death in most industrialized countries, and death at older ages is increasingly dominated by inflammation‐related diseases, the fact that allelic variation in *FOXO3* is now known to significantly modify this risk has important clinical implications, especially in high risk patient populations, such as those with type 2 diabetes (Gregg *et al*., 2014) and obesity (Olshansky *et al*., 2005). It has already become apparent that FoxO3 plays in important role in disease progression in age‐related diseases, such as cancer, and may be useful for monitoring responses to treatment (Monsalve & Olmos, 2011; Morris *et al*., 2015). Moreover, therapeutic interventions that modulate biological pathways associated with aging are now becoming a plausible approach to help people age more healthfully (Kennedy & Pennypacker, 2015). Our novel findings prompt consideration of *FOXO3* and its protein (Foxo3) as therapeutic targets in healthy aging with a focus on CHD and possibly other inflammation‐related diseases. More study is needed to determine molecular/cellular mechanisms.

## Experimental procedures

### Study design

We utilized data from the Kuakini Hawaii Lifespan Study, an ancillary study of the genetic and environmental antecedents of healthy aging and longevity, drawn from the Kuakini HHP cohort of aging men (Willcox *et al*., 2006, 2008; Donlon *et al*., 2012). The HHP began in 1965 as a population‐based prospective study of cardiovascular diseases and included 8006 American men of Japanese ancestry (Kagan, 1996). All study participants were recruited from World War II service records of 9877 men who had valid contact information, were born in 1900–1919, and who were living on the island of Oahu. For the current study, we utilized DNA samples and data collected in 1991–93, the first examination that focused on aging‐related measures, which were added at that time to the ongoing HHP study (White *et al*., 1996). We genotyped 3584 (~ 80% of available HHP survivors) stored blood samples collected at the 1991–1993 examination. Follow‐up monitoring total mortality and cause‐specific mortality for 17 years was conducted. All examinations, and the collection of biological samples, were approved by the Institutional Review Board of Kuakini Medical Center. Written informed consent was obtained at each examination from all study participants.

The Health ABC cohort was utilized for a replication study. Health ABC is a prospective observational study of 3075 well‐functioning, community‐dwelling, male and female, white and black Americans, aged 70–79 years at baseline, recruited from field centers at the University of Pittsburgh, Pennsylvania, and the University of Tennessee, Memphis, and followed up for 17 years.

### Genotyping

For HHP subjects, total cellular DNA was isolated from buffy coat of blood using the PureGene system (Gentra Systems, Inc., Big Lake, MN, USA) and quantified using PicoGreen staining (Molecular Probes, Eugene, OR, USA). We selected 32 SNPs that reflected the major genetic variability within intron 2 of *FOXO3*, based on degree of linkage disequilibrium and allele frequencies of ≥ 5%. We chose variant *rs2802292* (alleles *T*/*G*) as representative of the strongest longevity‐associated SNPs and genotyped an extended sample set of 3584 subjects. Genotyping for *rs2802292* involved an allelic discrimination assay.

TaqMan^®^ (Applied Biosystems, Inc. [ABI], Carlsbad, CA, USA) reagents (purchased from Life Technologies, Carlsbad, CA, USA) were used for PCR amplification under standard conditions with AmpliTaq Gold^®^ DNA polymerase (Perkin‐Elmer Corp., Waltham, MA, USA). PCR products were detected by TaqMan^®^ assay, using a 6‐FAM‐labeled FRET probe for one allele and a VIC‐labeled probe for the other allele, with minor groove binding (MGB) quenchers to enhance assay signal. PCR products were measured using an ABI Prism 7000 Sequence Detection System. Genotype data were managed through a sample processing and database system with previously proven reliability. All positive controls on each genotyping plate were evaluated for consistency. We have found that these methods are exceptionally robust and have an accuracy of > 99.9% on retesting and a success rate of 99.6% (data not shown). We regenotyped 1% of samples to monitor quality.

For Health ABC samples, genomewide SNP genotyping was performed by the Center for Inherited Disease Research using the Illumina Human1M‐Duo BeadChip system. Samples were excluded from the dataset for reasons of sample failure, genotypic sex mismatch, and first‐degree relative of an included individual based on genotype data. Health ABC genotyped 1794 self‐described white participants at baseline with available DNA and consent to genetics testing. Of these, 1661 passed quality control benchmarks (call rate > 97%, no sex mismatch, and cryptic relatedness). In addition, 1139 self‐described African Americans had genotype information available after quality control. Principal component analysis (PCA) was performed using Eigenstrat (Price *et al*., 2006). PCA was first performed on genotype data from all Health ABC participants and HapMap samples as anchor points to identify population outliers. PCA was then performed on genotype data separately for black and white participants who were not genetic outliers, and the resulting eigenvectors were used as covariates in regression models to adjust for population structure. Imputation was performed for the autosomes using MACH software version 1.0.16. SNPs with minor allele frequency ≥ 1%, call rate ≥ 97% and Hardy–Weinberg equilibrium *P *≥* *10^−6^ were used for imputation. HapMap II phased haplotypes were used as reference panels.

For whites, 914 263 SNPs passed quality control (QC) and were available for imputation using the HapMap CEPH reference panel (phase 2, release 22, build 36). For blacks, 1 007 948 SNPs passed QC and were available for imputation based on a 1:1 mixture of the CEPH:Yoruban (YRI) HapMap phase‐2 release‐22 build‐36 reference panel. A total of 2 543 887 SNPs in whites and 1 958 375 SNPs in blacks were imputed. SNP *rs2802292* was imputed. All fractional values within 0.1 of an integer were rounded to the nearest integer to create the number of *rs2802292* *G* alleles. The imputation quality measured by MACH *r* ^2^ was 0.99 in whites and blacks.

### Outcome measures

Total (all‐cause) mortality and cause‐specific mortality were the principal outcomes for the study. Death occurrence was ascertained by review of obituaries in local newspapers, through the medical examiner's office, by phone calls to family members or primary care physicians, and search of the National Death Index. Cause of death was determined by an expert morbidity and mortality committee, consisting of physicians who adjudicated the cause of death using standardized criteria, through continuous comprehensive surveillance of data collected from multiple sources (Kagan, 1996). These sources included death certificates, medical history from prior examinations, interview of healthcare proxies, hospital discharge records, cancer registry data, and autopsy reports, when available. Similar procedures were utilized in Health ABC.

### Statistics

Genotypes were evaluated and found to be in Hardy–Weinberg equilibrium. The relation of *FOXO3* genotype with total mortality and cause‐specific mortality was determined by Cox regression, adjusting for age, in carriers vs. noncarriers of the *G* allele of *rs2802292* in Japanese, whites, and blacks. For meta‐analysis of total mortality, age‐adjusted Cox regression models for Japanese, whites, and blacks were utilized, using both dominant and additive models for the effects of carrier status on total mortality. A dominant model was found to be most appropriate. Tests for heterogeneity were performed to assess suitability for a combined analysis. All statistical analyses were performed using the Statistical Analysis System (SAS) version 9.3 (SAS Institute, Cary, NC, USA).

## Funding

The work in Honolulu was funded by the Kuakini Medical Center, the US National Institutes of Health (contract N01‐AG‐4‐2149, Grants 5 U01 AG019349‐05, 5R01AG027060 [Kuakini Hawaii Lifespan Study], 5R01AG038707 [Kuakini Hawaii Healthspan Study]), contract N01‐HC‐05102 from the National Heart, Lung, and Blood Institute, and Kuakini Medical Center. We acknowledge the expert assistance of the Hawaii State Department of Health, particularly for supplying death certificate data. Research involving the Health ABC study was supported by National Institutes of Aging contracts N01AG62101, N01AG62103, and N01AG62106 and in part by the Intramural Research Program of the NIH, National Institute on Aging (Health ABC study). The Health ABC genomewide association study was funded by NIA grant 1R01AG032098‐01A1 to Wake Forest University Health Sciences, and genotyping services were provided by the Center for Inherited Disease Research (CIDR). CIDR is fully funded through a federal contract (number HHSN268200782096C) from the National Institutes of Health to The Johns Hopkins University. We thank all study participants and their families for their cooperation and the Hawaii State Department of Health for death certificate data. We thank Dr. Eunjung Lim for assistance with statistical analysis.

## Conflict of interest

None declared.

## Author contributions

BJW and TAD contributed to the study concept, data interpretation, study design, and writing the manuscript. KHM supervised recruitment and data collection on the HHP subjects and researchers at the Health ABC study sites recruited and characterized the Health ABC subjects. TAD and YL performed the HHP and Health ABC genotyping, respectively; MG, RA and PD performed cytokine analysis; TAD, YL, RC, EL, QH, NP, GJT, and DE managed data and contributed results; and RC, QH, YL, DES, and NP were responsible for data and statistical analyses. BJW, BJM, and TAD drafted the manuscript. All authors contributed to, read, and approved the final version of the manuscript.

## Supporting information


**Table S1.** Serum TNF‐α level by *FOXO3* *G* allele carrier status.

## References

[acel12452-bib-0001] Bao JM , Song XL , Hong YQ , Zhu HL , Li C , Zhang T , Chen W , Zhao SC , Chen Q (2014) Association between *FOXO3A* gene polymorphisms and human longevity: a meta‐analysis. Asian J. Androl. 16, 446–452. 24589462 10.4103/1008-682X.123673 PMC4023376

[acel12452-bib-0002] Blagosklonny MV (2010) Revisiting the antagonistic pleiotropy theory of aging: TOR‐driven program and quasi‐program. Cell Cycle 9, 3151–3156. 20724817 10.4161/cc.9.16.13120

[acel12452-bib-0003] Broer L , Buchman AS , Deelen J , Evans DS , Faul JD , Lunetta KL , Sebastiani P , Smith JA , Smith AV , Tanaka T , Yu L , Arnold AM , Aspelund T , Benjamin EJ , De Jager PL , Eirkisdottir G , Evans DA , Garcia ME , Hofman A , Kaplan RC , Kardia SL , Kiel DP , Oostra BA , Orwoll ES , Parimi N , Psaty BM , Rivadeneira F , Rotter JI , Seshadri S , Singleton A , Tiemeier H , Uitterlinden AG , Zhao W , Bandinelli S , Bennett DA , Ferrucci L , Gudnason V , Harris TB , Karasik D , Launer LJ , Perls TT , Slagboom PE , Tranah GJ , Weir DR , Newman AB , van Duijn CM , Murabito JM (2015) GWAS of longevity in CHARGE consortium confirms *APOE* and *FOXO3* candidacy. J. Gerontol. A Biol. Sci. Med. Sci. 70, 110–118. 25199915 10.1093/gerona/glu166 PMC4296168

[acel12452-bib-0004] Brooks‐Wilson AR (2013) Genetics of healthy aging and longevity. Hum. Genet. 132, 1323–1338. 23925498 10.1007/s00439-013-1342-z PMC3898394

[acel12452-bib-0501] Database of Single Nucleotide Polymorphisms (dbSNP). Bethesda (MD): National Center for Biotechnology Information, National Library of Medicine. dbSNP accession on 2/9/2016:{rs2802292}, (dbSNP Build ID: {100/146). Available from: http://www.ncbi.nlm.nih.gov/SNP/.

[acel12452-bib-0005] Dejean AS , Beisner DR , Ch'en IL , Kerdiles YM , Babour A , Arden KC , Castrillon DH , DePinho RA , Hedrick SM (2009) Transcription factor Foxo3 controls the magnitude of T cell immune responses by modulating the function of dendritic cells. Nat. Immunol. 10, 504–513. 19363483 10.1038/ni.1729 PMC2712214

[acel12452-bib-0006] Donlon TA , Curb JD , He Q , Grove JS , Masaki KH , Rodriguez B , Elliott A , Willcox DC , Willcox BJ (2012) FOXO3 gene variants and human aging: coding variants may not be key players. J. Gerontol. A Biol. Sci. Med. Sci. 67, 1132–1139. 22459618 10.1093/gerona/gls067 PMC3668389

[acel12452-bib-0007] Eijkelenboom A , Burgering BM (2013) FOXOs: signalling integrators for homeostasis maintenance. Nat. Rev. Mol. Cell Biol. 14, 83–97. 23325358 10.1038/nrm3507

[acel12452-bib-0008] Finch CE (2010) Evolution in health and medicine Sackler colloquium: evolution of the human lifespan and diseases of aging: roles of infection, inflammation, and nutrition. Proc. Natl. Acad. Sci. USA 107(Suppl 1), 1718–1724. 19966301 10.1073/pnas.0909606106 PMC2868286

[acel12452-bib-0009] Flachsbart F , Caliebe A , Kleindorp R , Blanché H , von Eller‐Eberstein H , Nikolaus S , Schreiber S , Nebel A (2009) Association of FOXO3A variation with human longevity confirmed in German centenarians. Proc. Natl. Acad. Sci. USA 106, 2700–2705. 19196970 10.1073/pnas.0809594106 PMC2650329

[acel12452-bib-0010] Franceschi C , Capri M , Monti D , Giunta S , Olivieri F , Sevini F , Panourgia MP , Invidia L , Celani L , Scurti M , Cevenini E , Castellani GC , Salvioli S (2007) Inflammaging and anti‐inflammaging: a systemic perspective on aging and longevity emerged from studies in humans. Mech. Ageing Dev. 128, 92–105. 17116321 10.1016/j.mad.2006.11.016

[acel12452-bib-0011] Fruman DA , Rommel C (2014) PI3K and cancer: lessons, challenges and opportunities. Nat. Rev. Drug Discov. 13, 140–156. 24481312 10.1038/nrd4204 PMC3994981

[acel12452-bib-0012] Gregg EW , Li Y , Wang J , Burrows NR , Ali MK , Rolka D , Williams DE , Geiss L (2014) Changes in diabetes‐related complications in the United States, 1990–2010. N. Engl. J. Med. 370, 1514–1523. 24738668 10.1056/NEJMoa1310799

[acel12452-bib-0013] Kagan A , ed. (1996) The Honolulu Heart Program: An Epidemiological Study of Coronary Heart Disease and Stroke. Amsterdam, The Netherlands: Harwood Academic Publishers.

[acel12452-bib-0014] Kennedy BK , Pennypacker JK (2015) Aging interventions get human. Oncotarget 6, 590–591. 25612254 10.18632/oncotarget.3173 PMC4359240

[acel12452-bib-0015] Kenyon CJ (2010) The genetics of ageing. Nature 464, 504–512. 20336132 10.1038/nature08980

[acel12452-bib-0016] Kuningas M , Mägi R , Westendorp RG , Slagboom PE , Remm M , van Heemst D (2007) Haplotypes in the human Foxo1a and Foxo3a genes; impact on disease and mortality at old age. Eur. J. Hum. Genet. 15, 294–301. 17245409 10.1038/sj.ejhg.5201766

[acel12452-bib-0017] Lam EW , Brosens JJ , Gomes AR , Koo CY (2013) Forkhead box proteins: tuning forks for transcriptional harmony. Nat. Rev. Cancer 13, 482–495. 23792361 10.1038/nrc3539

[acel12452-bib-0018] Lee JC , Espéli M , Anderson CA , Linterman MA , Pocock JM , Williams NJ , Roberts R , Viatte S , Fu B , Peshu N , Hien TT , Phu NH , Wesley E , Edwards C , Ahmad T , Mansfield JC , Gearry R , Dunstan S , Williams TN , Barton A , Vinuesa CG , Parkes M , Lyons PA , Smith KG (2013) Human SNP links differential outcomes in inflammatory and infectious disease to a FOXO3‐regulated pathway. Cell 155, 57–69. 24035192 10.1016/j.cell.2013.08.034 PMC3790457

[acel12452-bib-0019] Miyamoto K , Araki KY , Naka K , Arai F , Takubo K , Yamazaki S , Matsuoka S , Miyamoto T , Ito K , Ohmura M , Chen C , Hosokawa K , Nakauchi H , Nakayama K , Nakayama KI , Harada M , Motoyama N , Suda T , Hirao A (2007) Foxo3a is essential for maintenance of the hematopoietic stem cell pool. Cell Stem Cell 1, 101–112. 18371339 10.1016/j.stem.2007.02.001

[acel12452-bib-0020] Monsalve M , Olmos Y (2011) The complex biology of FOXO. Curr. Drug Targets 12, 1322–1350. 21443460 10.2174/138945011796150307

[acel12452-bib-0021] Morris BJ , Willcox DC , Donlon TA , Willcox BJ (2015) FOXO3: a major gene for human longevity – A Mini‐Review. Gerontology 61, 515–525. 25832544 10.1159/000375235 PMC5403515

[acel12452-bib-0502] Morris BJ , Chen R , Donlon TA , Evans DS , Tranah GJ , Parimi N , Ehret GB , Newton‐Cheh C , Seto T , Willcox DC , Masaki KH , Kamide K , Ryuno H , Oguro R , Nakama C , Kabayama M , Yamamoto K , Sugimoto K , Ikebe K , Masui Y , Arai Y , Ishizaki T , Gondo Y , Rakugi H (2016) Association analysis of *FOXO3* longevity variants with blood pressure and essential hypertension. Am. J. Hypertens. (Epub ahead of print Oct 16, 2015) 10.1093/ajh/hpv171 PMC5055732 26476085

[acel12452-bib-0022] Murabito JM , Yuan R , Lunetta KL (2012) The search for longevity and healthy aging genes: insights from epidemiological studies and samples of long‐lived individuals. J. Gerontol. A Biol. Sci. Med. Sci. 67, 470–479. 22499766 10.1093/gerona/gls089 PMC3326242

[acel12452-bib-0023] National Institute on Aging . (2007) Living Long and Well in the 21st Century Strategic Directions for Research on Aging: National Institute on Aging [WWW document]. URL http://www.nia.nih.gov/about/living-long-well-21st-century-strategic-directions-research-aging [accessed on 20 December 2014].

[acel12452-bib-0024] Oh HM , Yu CR , Golestaneh N , Amadi‐Obi A , Lee YS , Eseonu A , Mahdi RM , Egwuagu CE (2011) STAT3 protein promotes T‐cell survival and inhibits interleukin‐2 production through up‐regulation of class O forkhead transcription factors. J. Biol. Chem. 286, 30888–30897. 21730069 10.1074/jbc.M111.253500 PMC3162449

[acel12452-bib-0025] Olshansky SJ , Passaro DJ , Hershow RC , Layden J , Carnes BA , Brody J , Hayflick L , Butler RN , Allison DB , Ludwig DS (2005) A potential decline in life expectancy in the United States in the 21st century. N. Engl. J. Med. 352, 1138–1145. 15784668 10.1056/NEJMsr043743

[acel12452-bib-0026] Pawlikowska L , Hu D , Huntsman S , Sung A , Chu C , Chen J , Joyner AH , Schork NJ , Hsueh WC , Reiner AP , Psaty BM , Atzmon G , Barzilai N , Cummings SR , Browner WS , Kwok PY , Ziv E (2009) Association of common genetic variation in the insulin/IGF1 signaling pathway with human longevity. Aging Cell 8, 460–472. 19489743 10.1111/j.1474-9726.2009.00493.x PMC3652804

[acel12452-bib-0027] Peng SL (2007) Immune regulation by Foxo transcription factors. Autoimmunity 40, 462–469. 17729040 10.1080/08916930701464913

[acel12452-bib-0028] Price AL , Patterson NJ , Plenge RM , Weinblatt ME , Shadick NA , Reich D (2006) Principal components analysis corrects for stratification in genome‐wide association studies. Nat. Genet. 38, 904–909. 16862161 10.1038/ng1847

[acel12452-bib-0029] Salminen A , Huuskonen J , Ojala J , Kauppinen A , Kaarniranta K , Suuronen T (2008) Activation of innate immunity system during aging: NF‐kB signaling is the molecular culprit of inflamm‐aging. Ageing Res. Rev. 7, 83–105. 17964225 10.1016/j.arr.2007.09.002

[acel12452-bib-0030] Shadyab AH , LaCroix AZ (2015) Genetic factors associated with longevity: a review of recent findings. Ageing Res. Rev. 19, 1–7. 25446805 10.1016/j.arr.2014.10.005

[acel12452-bib-0031] Shen J , Tower J (2010) Drosophila foxo acts in males to cause sexual‐dimorphism in tissue‐specific p53 life span effects. Exp. Gerontol. 45, 97–105. 19840842 10.1016/j.exger.2009.10.009 PMC2814947

[acel12452-bib-0033] Soerensen M , Nygaard M , Dato S , Stevnsner T , Bohr VA , Christensen K , Christiansen L (2015) Association study of *FOXO3A* SNPs and aging phenotypes in Danish oldest‐old individuals. Aging Cell 14, 60–66. 25470651 10.1111/acel.12295 PMC4326903

[acel12452-bib-0034] Takata H , Suzuki M , Ishii T , Sekiguchi S , Iri H (1987) Influence of major histocompatibility complex region genes on human longevity among Okinawan‐Japanese centenarians and nonagenarians. Lancet 2, 824–826. 2889033 10.1016/s0140-6736(87)91015-4

[acel12452-bib-0035] US Department of Health and Human Services . (2012) Office of Disease Prevention and Health Promotion. Healthy People 2020 [WWW document]. URL. http://www.healthypeople.gov [accessed on 20 December 2014].

[acel12452-bib-0036] White L , Petrovitch H , Ross GW , Masaki KH , Abbott RD , Teng EL , Rodriguez BL , Blanchette PL , Havlik RJ , Wergowske G , Chiu D , Foley DJ , Murdaugh C , Curb JD (1996) Prevalence of dementia in older Japanese‐American men in Hawaii: the Honolulu‐Asia Aging Study. JAMA 276, 955–960. 8805729

[acel12452-bib-0037] Willcox BJ , He Q , Chen R , Yano K , Masaki KH , Grove JS , Donlon TA , Willcox DC , Curb JD (2006) Midlife risk factors and healthy survival in men. JAMA 296, 2343–2350. 17105797 10.1001/jama.296.19.2343

[acel12452-bib-0038] Willcox BJ , Donlon TA , He Q , Chen R , Grove JS , Yano K , Masaki KH , Willcox DC , Rodriguez B , Curb JD (2008) FOXO3A genotype is strongly associated with human longevity. Proc. Natl. Acad. Sci. USA 105, 13987–13992. 18765803 10.1073/pnas.0801030105 PMC2544566

[acel12452-bib-0039] Yang JY , Xia W , Hu MC (2006) Ionizing radiation activates expression of FOXO3a, Fas ligand, and Bim, and induces cell apoptosis. Int. J. Oncol. 29, 643–648. 16865280 PMC2632978

[acel12452-bib-0040] Zhao Y , Yu Y , Tian X , Yang X , Li X , Jiang F , Chen Y , Shi M (2014) Association study to evaluate FoxO1 and FoxO3 gene in CHD in Han Chinese. PLoS One 9, e86252. 24489705 10.1371/journal.pone.0086252 PMC3904908

