# Supplementary material for: The FoxO3 gene and cause‐specific mortality
Source: Aging Cell. 2016 Apr 13;15(4):617–24. doi: 10.1111/acel.12452 (PMC4933667; doi:10.1111/acel.12452)
Supplement: Supplementary file 1 — Table S1. Serum TNF‐α level by FOXO3 G allele carrier status. [file ACEL-15-617-s001.docx]

**SUPPLEMENTARY MATERIAL**

**Supplementary Table 1. Serum TNF-α level by *FOXO3* *G* allele carrier status**

| **Variable** | ***TT*** | ***GT*/*GG*** | ***P*** |
| --- | --- | --- | --- |
| **n** | 55 | 66 |  |
| Age (mean ± SD) | 59.2 ± 4.9 | 61.4 ± 6.1 | 0.014 |
| TNF-α (mean ± SE) | 4.2 ± 0.48 | 2.9 ± 0.30 | 0.018 |
| Log TNF-α (± SE) | 1.1 ± 0.12 | 0.69 ± 0.11 | 0.018 |
